# A Novel Single-Cell RNA Sequencing Data Feature Extraction Method Based on Gene Function Analysis and Its Applications in Glioma Study

**DOI:** 10.3389/fonc.2021.797057

**Published:** 2021-11-30

**Authors:** Jujuan Zhuang, Changjing Ren, Dan Ren, Yu’ang Li, Danyang Liu, Lingyu Cui, Geng Tian, Jiasheng Yang, Jingbo Liu

**Affiliations:** ^1^ School of Science, Dalian Maritime University, Dalian, Liaoning, China; ^2^ Pathology Department, Da Qing Long Nan Hospital, Qiqihar Medical University, Heilongjiang, China; ^3^ Maths and Applied Mathematics, University of Nottingham, Nottingham, United Kingdom; ^4^ Geneis (Beijing) Co., Ltd., Beijing, China; ^5^ School of Electrical and Information Engineering, Anhui University of Technology, Anhui, China

**Keywords:** single-cell RNA sequencing, GO enrichment analysis, KPCA, semantic similarity analysis, Gene Ontology

## Abstract

Critical in revealing cell heterogeneity and identifying new cell subtypes, cell clustering based on single-cell RNA sequencing (scRNA-seq) is challenging. Due to the high noise, sparsity, and poor annotation of scRNA-seq data, existing state-of-the-art cell clustering methods usually ignore gene functions and gene interactions. In this study, we propose a feature extraction method, named FEGFS, to analyze scRNA-seq data, taking advantage of known gene functions. Specifically, we first derive the functional gene sets based on Gene Ontology (GO) terms and reduce their redundancy by semantic similarity analysis and gene repetitive rate reduction. Then, we apply the kernel principal component analysis to select features on each non-redundant functional gene set, and we combine the selected features (for each functional gene set) together for subsequent clustering analysis. To test the performance of FEGFS, we apply agglomerative hierarchical clustering based on FEGFS and compared it with seven state-of-the-art clustering methods on six real scRNA-seq datasets. For small datasets like Pollen and Goolam, FEGFS outperforms all methods on all four evaluation metrics including adjusted Rand index (ARI), normalized mutual information (NMI), homogeneity score (HOM), and completeness score (COM). For example, the ARIs of FEGFS are 0.955 and 0.910, respectively, on Pollen and Goolam; and those of the second-best method are only 0.938 and 0.910, respectively. For large datasets, FEGFS also outperforms most methods. For example, the ARIs of FEGFS are 0.781 on both Klein and Zeisel, which are higher than those of all other methods but slight lower than those of SC3 (0.798 and 0.807, respectively). Moreover, we demonstrate that CMF-Impute is powerful in reconstructing cell-to-cell and gene-to-gene correlation and in inferring cell lineage trajectories. As for application, take glioma as an example; we demonstrated that our clustering methods could identify important cell clusters related to glioma and also inferred key marker genes related to these cell clusters.

## Introduction

Biological tissues are composed of a variety of heterogeneous cells, and their presence will have a profound impact on the biological functions of cells. The single-cell RNA sequencing (scRNA-seq) technology (1) allows for the analysis of gene expression data at the level of individual cells. As a promising tool, scRNA-seq technology can reveal heterogeneity among cells and identify new putative cell types and cell states (2–5). Cell clustering is the main approach for cell type and cell state inference. Despite the rapid development of scRNA-seq technology, the biological fluctuation and protocol technical biases in single-cell experiments and the high dimensionality and sparsity of scRNA-seq data make cell clustering based on scRNA-seq challenging (6).

Various scRNA-seq clustering methods have been developed in recent years, most of which are based on similarity measurement between cells. For example, CORR derives cell similarity in genetic differences between cell pairs (7). SIMLR adopts multiple Gaussian kernel representations, which allows greater flexibility than a single kernel or similarity measures in defining cell-to-cell similarities (8). Seurat constructs weighted nearest neighbor graph based on typical correlation to obtain technology similarity between cells (9). SC3 constructs a consensus similarity matrix based on three measurements of distances (10). SSC (11), SSSC (12), and S3C2 (13) are sparse subspace clustering methods, which aim to describe the relations among all elements as a combination in the same subspace rather than consider pair elements only. Most of the scRNA-seq cell clustering methods derive the similarity between cell pairs by considering the complete gene expression matrix, which ignore the function of genes on cell clustering from the perspective of molecular mechanism and the impact of biological significance. Since the differences in the morphology and structure of different cells are caused by the selective expression of genes, it is more reasonable to analyze scRNA-seq data in terms of functional gene sets.

The Gene Ontology (GO) (14, 15) is a formal representation of a body of knowledge within biological domain, which consists of a set of gene classes with relations that operate between them. It describes the biological knowledge of gene and gene product with respect to three aspects: the molecular functions (MFs), cellular locations, and processes that gene products may carry out. It stands to reason that different types of cells may have different gene expression characteristics in a GO term gene set.

**Figure 1:**
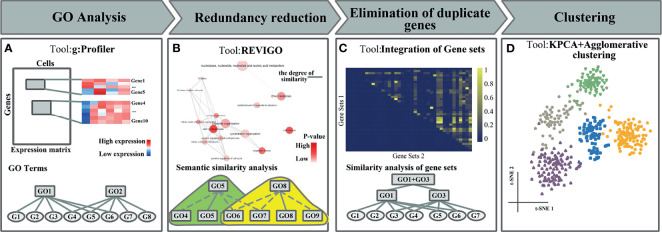
The flowchart of FEGFS + clustering. **(A)** Gene Ontology (GO) analysis. **(B)** Redundancy reduction. **(C)** Elimination of duplicate genes. **(D)** Feature extraction and clustering analysis.

In this work, we propose a feature extraction method based on gene functional sets, named FEGFS, to analyze and integrate the gene expression characteristics of cells on different functional gene sets derived from GO terms (**Figure 1**). We select functional gene sets by gene functional enrichment analysis, and the terms semantic similarity analysis and multistep integration of gene sets for scRNA-seq data, and kernel principal component analysis (KPCA) is applied on the single-cell gene expression data of these selected gene functional sets to reduce the dimension of features, and the reduced expression data are integrated into a feature matrix. We consider cell clustering in terms of feature matrix rather than using the expression values of all genes as a whole in scRNA-seq analysis, which not only conforms to biological rules more but also can improve the cell clustering effect. To evaluate the performance of FEGFS, we use agglomerative hierarchical clustering for cell clustering on the derived feature matrix, and we compared the clustering results with seven state-of-art clustering methods on six independent datasets, and the results demonstrate that FEGFS can significantly improve clustering accuracy.

## Method

### Datasets and Data Preprocessing

We adopt six real scRNA-seq datasets in this study to evaluate the performance of FEGFS. The cell labels in each scRNA-seq dataset are known or valid in their respective studies, the sample labels of Zeisel dataset are predicted according to the experiment (16), and the sample labels of the other five datasets are obtained from experimental studies. These datasets are grouped into two levels (small sample (with number of samples ≤1,000) and large sample [with number of samples >1,000)] according to the number of cells. Pollen (17), Biase (18), Goolam (19), and Patel (20) datasets are assigned to small sample datasets. Klein (21) and Zeisel (22) datasets are assigned to large sample datasets. We summarize the details of the six real scRNA-seq datasets (**Table 1**). As shown in the table, the numbers of samples of these datasets range from 56 (Biase) to 3005 (Zeisel); and the numbers of cell types range from 4 (Klein) to 11 (Pollen). During our downstream analysis, the proportion of principal components retained by feature extraction of different levels datasets is also different.

**Table 1:** A summary of six scRNA-seq datasets used in this study.

Datasets	Cell types	Number of cells	Number of GO terms	Number of genes	Units	Organism
*Biase*	4	56	201	22,528	FPKM	*Mus musculus*
*Goolam*	5	124	213	22,624	Count	*M. musculus*
*Pollen*	11	301	282	13,678	TPM	*Homo sapiens*
*Patel*	5	430	208	5,610	TPM	*H. sapiens*
*Klein*	4	2717	237	22,192	UMI	*M. musculus*
*Zeisel*	7	3005	253	11,713	UMI	*M. musculus*

scRNA-seq, single-cell RNA sequencing; GO, Gene Ontology; FPKM, fragments per kilobase of transcript per million mapped reads; TPM, transcripts per kilobase million; UMI, unique molecular identifier.

In order to eliminate the interference with noise genes in scRNA-seq datasets, the actual number of noise genes removed from the datasets is determined by the number of samples of the datasets. In this study, we adopt 3 Units multiplied by 1% of the number of samples in the dataset to remove noise genes (e.g., in Pollen dataset (with number of cells of 301): 3 Units × 3 (1% of samples) = 9 Units; that is, when the gene is expressed in less than nine cells, the gene is removed) (23), and the gene expression values are log-transformed with pseudo-count 1:


(1) $$F(X)=\log_{10}(X+1)$$

### Gene Ontology Enrichment Analysis

GO is a widely used biological database. It consists of two aspects: one is the GO itself; that is, the terms defined by biologists and the structural relationships between them. The other is the annotation of GO, which is the relationship between gene products and the entries. As a strictly functional category, GO links the relationships between different functional categories by directed acyclic graphs (DAGs).

We use g:Profiler (24) to characterize and process the list of genes in the scRNA-seq dataset. Before processing, we first apply g:Convert to transform gene identifier into the internal format of Ensemble genes. Then we apply g:GOSt to analyze the gene table of various organisms. The algorithm is based on the gene set structure of biological term annotation. The purpose is to distinguish meaningful and meaningless biological results, reduce the importance of p-value, and eliminate the false-positive problem. Statistical enrichment analysis maps genes to known functional information sources (Biological Process (BP), Cellular Component (CC), and MF) and detects and counts the significantly rich GO nodes.

### Reduce the Redundancy of Gene Ontology Term Set

In order to alleviate the redundancy of GO term sets, we apply REVIGO (25) to perform semantic similarity analysis. SimRel as the semantic similarity measure for comparison is defined (26) as follows:


(2) $$sim(g_{1},g_{2})=\frac{2\log P(MIA)}{\log P(g_{1})+\log P(g_{2})}(1-P(MIA))$$

where *g*_1_ and *g*_2_ are two GO terms, P() is the relative frequency of GO Term in UniProt database, *MIA* ∈ *S*(*g*_1_, *g*_2_), and *S*(*g*_1_, *g*_2_) is the common ancestor set of terms *g*_1_ and *g*_2_ in the ontology.

The p-value of each GO term that is used in function enrichment analysis and subsequent semantic similarity analysis is defined as follows:


(3) $$P(X=k)=\frac{\left(\begin{array}{c} M\\ k \end{array}\right)\left(\begin{array}{c} N-M\\ n-k \end{array}\right)}{\left(\begin{array}{c} N\\ n \end{array}\right)}$$

where N is the number of genes in the genome that belong to the same GO level (BP, MF and CC) with considered GO term; M is the number of genes of this GO term; n is the number of genes in our input data that belong to the same GO level (BP, MF, and CC) with this GO term; and k is the number of genes in our input data that belong to the GO term.

The calculated p-value is corrected by false discovery rate (FDR) (27). In our test, we choose *FDR* = 0.05 as the threshold. GO nodes with *FDR* ≤ 0.05 are defined as significantly enriched nodes.

In order to reduce the redundancy of GO term set, we apply REVIGO to select the representative GO term for each cluster according to p-values. After the semantic similarity analysis, there are about 100–250 GO nodes in GO term set, in which gene duplication problems are very serious.

To further reduce redundancy, the gene repetitive rate matrix of GO nodes is constructed, and the calculation formula of each element in the matrix is as follows:


(4) $$\begin{array}{l} R_{i,j}=\frac{N(GO_{i},GO_{j})}{M(GO_{i},GO_{j})}\\ with\\ M(GO_{i},GO_{j})=\min\{\begin{array}{cc} gene\_num\{GO_{i}\}, & gene\_num\{GO_{j}\} \end{array}\}\\ N(GO_{i},GO_{j})=gene\_num\{GO_{i}\cap GO_{j}\} \end{array}$$

where *gene*_*num*{*GO_s_*} represents the number of genes in GO nodes.

The gene repetitive rate matrix is applied to merge the GO terms. Specifically, if the elements of one GO term belong to another, GO terms containing a larger number of genes are retained, while GO terms containing a smaller number are deleted; then, with 0.8 as the threshold of repetitive rate, GO terms in pairs are merged into a new terms set so as to greatly reduce the redundancy of GO terms set, and the scRNA-seq expression matrix restricted on each new term is named as functional feature matrix.

### Feature Extraction and Cluster Analysis

In the process of feature extraction, after comparing several dimension reduction methods—t-distributed stochastic neighbor embedding (t-SNE) (28), multidimensional scaling (MDS), and KPCA—we choose KPCA as our feature extraction method. KPCA is a non-linear feature dimension reduction algorithm to process linear inseparable dataset, in which a non-linear mapping is used to map the samples in the input matrix X to a high-dimensional or even infinite-dimensional space (called feature space) such that the samples are linearly separable in feature space, and then PCA is applied to reduce the dimension in the high-dimensional space.

In our study, we compare several kernel methods (radial basis function, sigmoid, cosine etc.), and we choose cosine kernel method of KPCA in the data dimension reduction of functional feature matrices. The cosine kernel function is shown as follows:


(5) $$\kappa (x_{i},x_{j})=\frac{\phi (x_{i})\phi {\left(x_{j}\right)}^{T}}{\|\phi (x_{i})\|\left\|\phi (x_{j})\right\|}$$

Agglomerative hierarchical clustering method is applied on the reduced functional feature matrices, and we evaluate the clustering performance by adjusted Rand index (ARI) and normalized mutual information (NMI). By calculating the distances of sample set pairs, agglomerative hierarchical clustering merges the two sample sets with the minimum distance and repeats the above process by recalculating the distances of the new sample sets pairs. The distance of sample set pair is calculated by Euclidean distance D:


(6) $$D_{i,j}=\sqrt{{\left(x_{i}-y_{i}\right)}^{2}+{\left(x_{j}-y_{j}\right)}^{2}}$$

### Evaluation Measures

In order to evaluate the effectiveness of functional feature matrix for cell clustering, we choose NMI, ARI, homogeneity score (HOM), and completeness score (COM) to quantify the consistency between the inferred and predefined cell clusters in each scRNA-seq data.

ARI is defined as follows:


(7) $$ARI=\frac{RI‐E[RI]}{\max(RI)-E[RI]}$$

(8) $$RI=\frac{F+G}{\left(\begin{array}{c} N\\ 2 \end{array}\right)}$$

where F is the number of pair samples in the same category in both the real label and the clustering prediction label, while G is the number of pair samples in different categories. N is the number of samples in the dataset.

NMI is defined as follows:


(9) $$NMI=\frac{2I(F,G)}{H(F)+H(G)}$$

where *I*(*F*,*G*) is the mutual information of *F* and *G*


(10) $$I(F,G)=-\sum_{i=1}^{k}\sum_{j=1}^{k}\frac{\left|F_{i}\cap G_{j}\right|}{N}\log\frac{N\left|F_{i}\cap G_{j}\right|}{\left|F_{i}\right|\times \left|G_{j}\right|}$$

*H*(*F*) and *H*(*G*) are the entropy of partitions *F* and *G*; *F_i_* is the dataset belonging to class i; and *G_j_* is the dataset belonging to class j in the clustering results.


(11) $$\begin{array}{c} H(F)=-\sum_{i=1}^{k}\frac{F_{i}}{N}\log\frac{F_{i}}{N}\\ H(G)=-\sum_{j=1}^{k}\frac{G_{j}}{N}\log\frac{G_{j}}{N} \end{array}$$

where N is the total number of cells.

HOM is defined as


(12) $$HOM=\frac{1}{k}\sum_{i=1}^{k}\frac{N(F_{i},G_{i})}{N(G_{i})}$$

COM is defined as


(13) $$COM=\frac{1}{k}\sum_{i=1}^{k}\frac{N(F_{i},G_{i})}{N(F_{i})}$$

where *N*(*F_i_*, *G_i_*) is the number of samples correctly classified in the ith cluster, and *N*(*G_i_*) is the total number of samples in the ith cluster. *N*(*F_i_*) is the total number of samples in the ith type.

### Software Availability

FEGFS is implemented in Python3 as an open-source software under the GNU General Public License, and the source code is freely available together with full documentation at https://github.com/R-c-j/FEGFS.

## Result

### The Construction Principle of Functional Feature Matrix

The construction of functional feature matrix is mainly divided into three steps: GO functional enrichment analysis, and GO term sets redundancy reduction and feature extraction.

In the process of GO functional enrichment analysis, the genes of real scRNA-seq data are used as the input set when statistical enrichment analysis is performed according to their molecular mechanisms. According to their MFs, cell environment, and the BPs that they participate in, the genes are divided into three types, MF, CC, and BP; taking Pollen dataset as an example, it contains 13,678 genes after preprocessing, and the ordered query is used in the functional enrichment analysis (g:Profiler), with the default options: User threshold is 0.05 and Significance threshold is G:SCS, and we get 800 GO nodes after the functional enrichment analysis.

The number of GO nodes obtained from the statistical enrichment analysis is huge (about 1,000), and the redundancy is high. We perform semantic similarity analysis on the GO term set to remove the redundant nodes by REVIGO (25), in which we choose the father GO node as the representative node in each cluster with SimRel equals 0.4. After semantic similarity analysis, the number of GO nodes is about 200 to 300, and there are many duplicates of the genes between some GO nodes. To solve this problem, we calculate the repetitive rate for any two GO nodes, and we construct gene repetitive rate matrix, which is symmetric. We preliminarily filter the completely covered GO nodes, take 0.8 as the threshold of repetitive rate and merge the nodes, and recalculate the gene repetitive rate and repeat the above process. After screening twice, the number of nodes in the GO term set reduces to about 80–150. For example, in Pollen dataset, after GO functional enrichment analysis and semantic similarity analysis, GO:0033554 (cellular response to stress) contains 810 genes, GO:0070498 (interleukin-1-mediated signaling pathway) contains 46 genes, and the gene repetitive rate between the two GO terms is 1, so GO:0070498 is filtered and GO:0033554 is reserved. After all GO nodes with gene repetitive rate of 1 are filtered, GO terms are screened twice with the gene repetitive rate of 0.8; for example, GO:0045202 (synapse) contains 10 genes, GO:0048519 (negative regulation of BP) contains 119 genes, there are nine genes in the intersection of the two terms, and the repetitive rate is 0.9 > 0.8, so GO:0045202 and GO:0048519 are combined into a new functional feature node.

**Figure 2:**
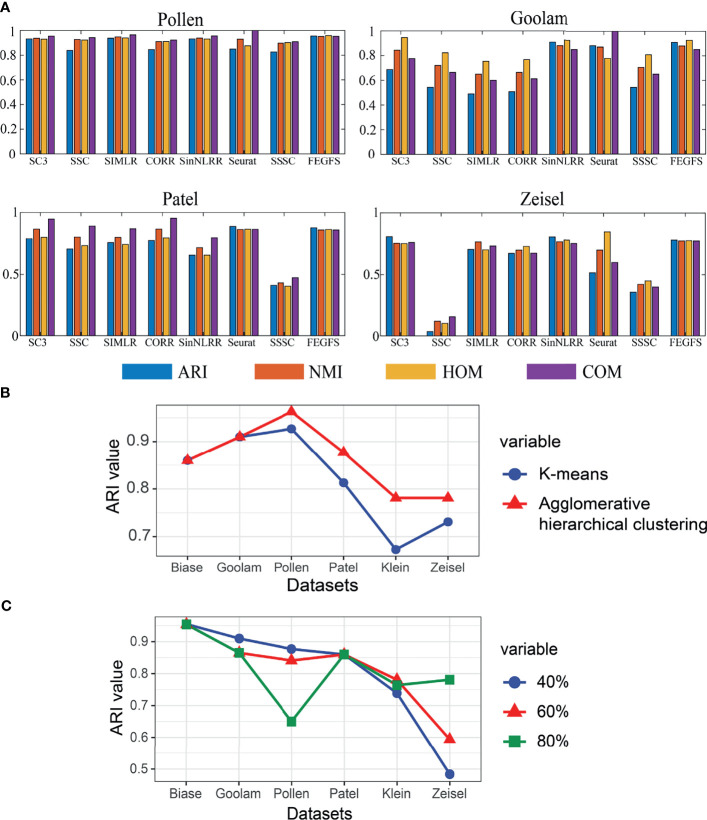
**(A)** The clustering effect comparison of SC3, SSC, SIMLR, CORR, SinNLRR, Seurat, SSSC, and S3C2 on four datasets. **(B)** Line graph of retention rate based on adjusted Rand index (ARI). **(C)** Comparison of ARI values between K-means and agglomerative hierarchical clustering based on FEGFS.

We perform feature extraction on each functional feature matrix by applying KPCA. The proportion of principal components to be retained is different according to different levels of sample sets (**Figure 2B**). In the four small sample datasets of Pollen, Goolam, Patel, and Biase [*num_sample_* ∈ (0,1000)], we choose 40% principal component retention ratio; and in larger sample datasets, such as Klein dataset [*num_sample_* ∈ (1000,3000], we choose 60% principal component retention ratio, and in datasets with a sample size greater than 3,000, such as Zeisel dataset [*num_sample_* ∈ (1000,3000)], we use 80% of the principal component retention ratio to reduce the dimension of gene expression matrix of each functional feature matrix.

After the above three steps of processing, we integrate all the processed functional feature matrices into a feature matrix and use it for downstream analysis (**Figure 1**).

### Clustering Effect Evaluation

**Figure 3:**
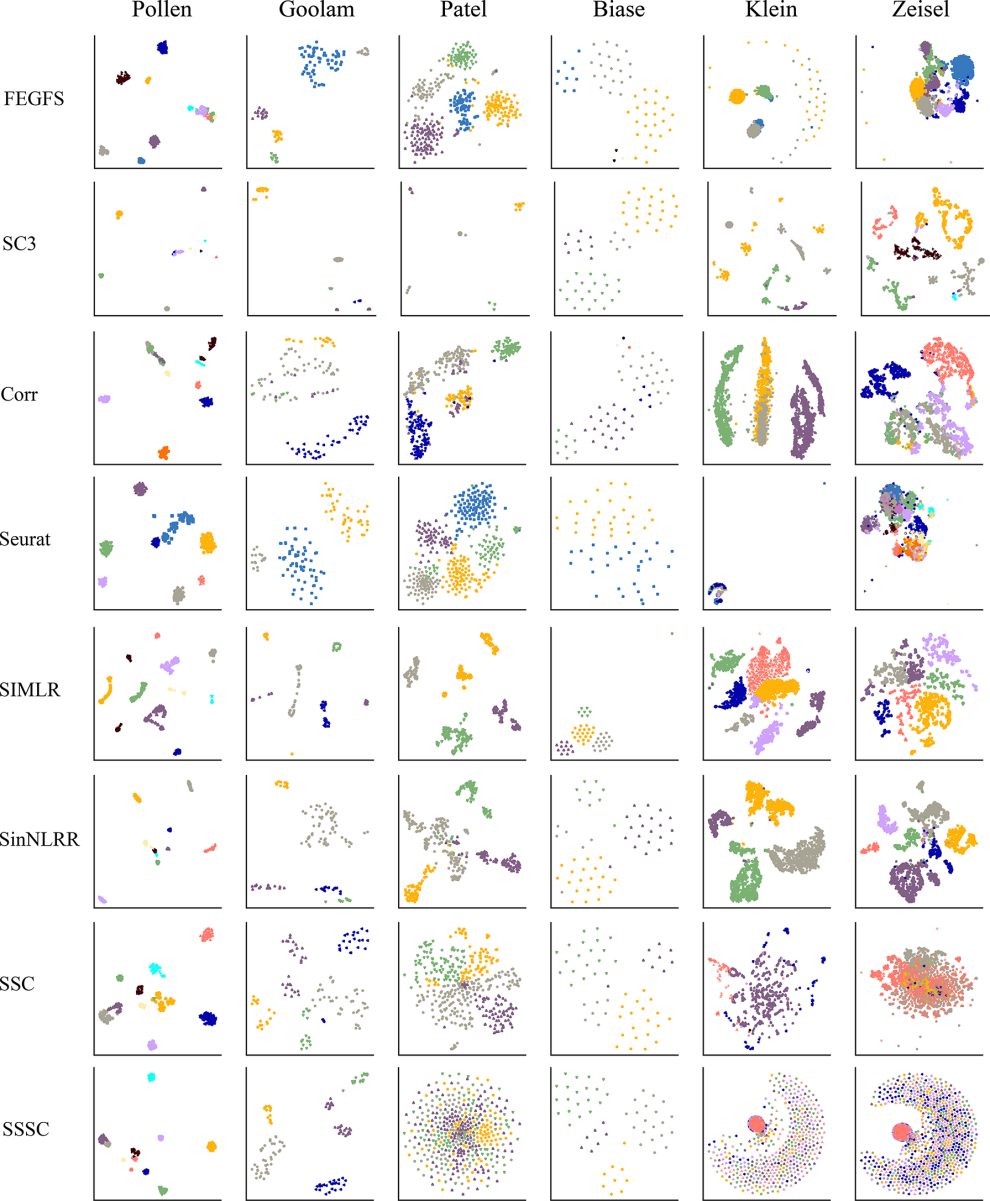
The t-distributed stochastic neighbor embedding (t-SNE) visualization of cells on six real single-cell RNA sequencing (scRNA-seq) datasets using different clustering methods.

We evaluate the performance of FEGFS on six real scRNA-seq datasets by cell clustering and visualizing with t-SNE, where cells were colored according to their cell type annotations (**Figure 3**). In our work, we apply agglomerative hierarchical clustering on these six datasets.

To prove the effectiveness of FEGFS, we compare the results of agglomerative hierarchical clustering with other seven state-of-the-art clustering methods (**Figure 2A**), including SC3 (10), Seurat (9), CORR (7), SIMLR (8), SSSC (12), SinNLRR (29), and SSC (11).

**Figure 4:**
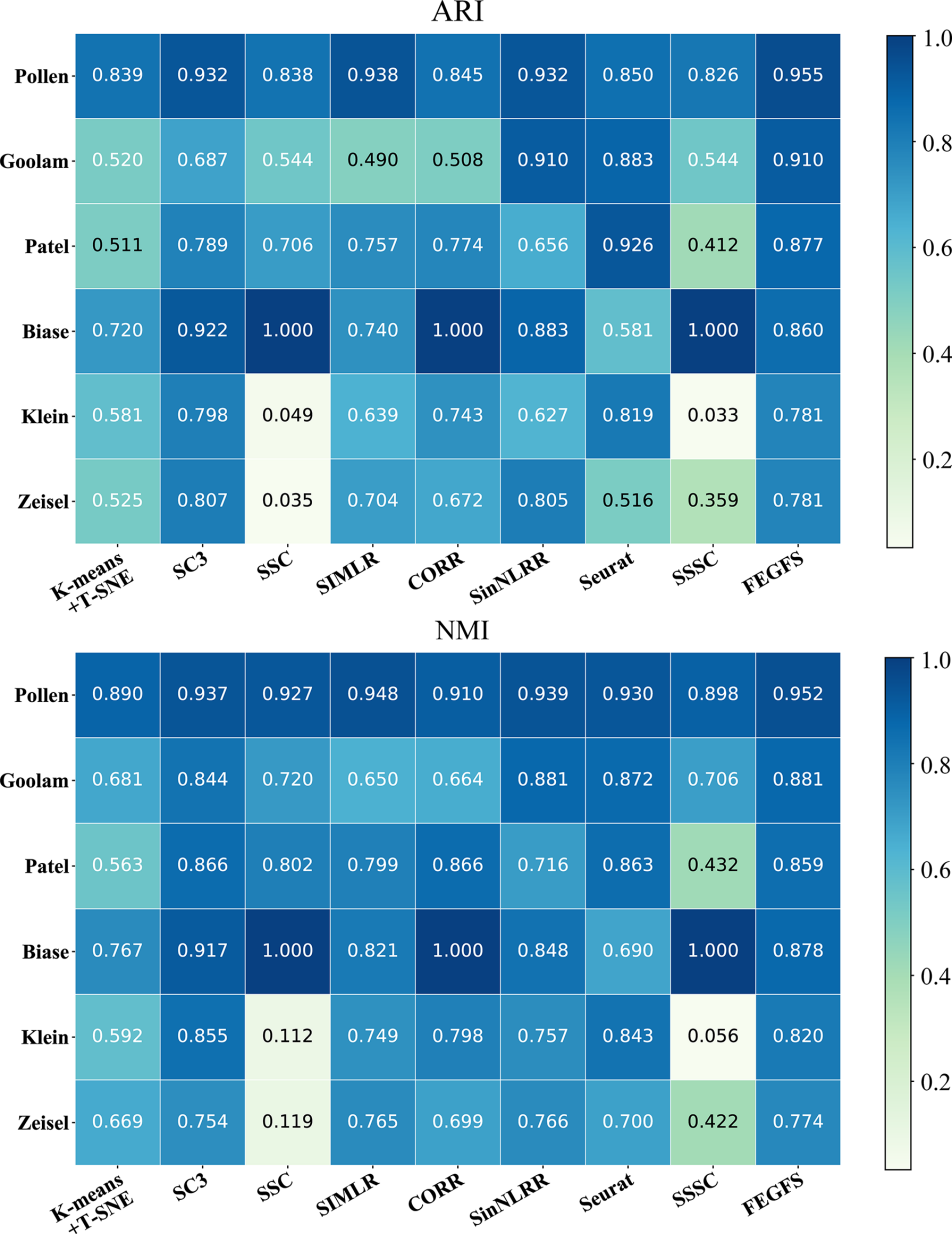
Gene Functional Network significantly improves the performance of the existing cell type identification tool Agglomerative. The adjusted Rand index (ARI) and normalized mutual information (NMI) obtained by clustering on six scRNA-seq datasets using different cluster algorithms.

In the process of comparison, all of the other methods use the same data preprocessing method as FEGFS. With the four evaluation indicators ARI, NMI, HOM, and COM, the clustering results of all methods on the six scRNA-seq datasets are shown in **Figure 4**, and the results of k-means clustering based on function feature matrix are shown in **Figure 2C**. Compared with SSC and its improved methods SSSC, our method is significantly superior in scRNA-seq datasets of Pollen, Goolam, Patel, Klein, and Zeisel. For the small sample datasets Pollen and Goolam, the highest ARI values (0.955 and 0.910) are obtained by FEGFS; even in the two larger sample datasets Zeisel and Klein, our method is only second among all the algorithms to SC3. Therefore, FEGFS can help to extract the characteristics of different cell types and promote the analysis of single-cell transcriptome data.

### Expression Distribution of Cluster Marker Genes

**Figure 5:**
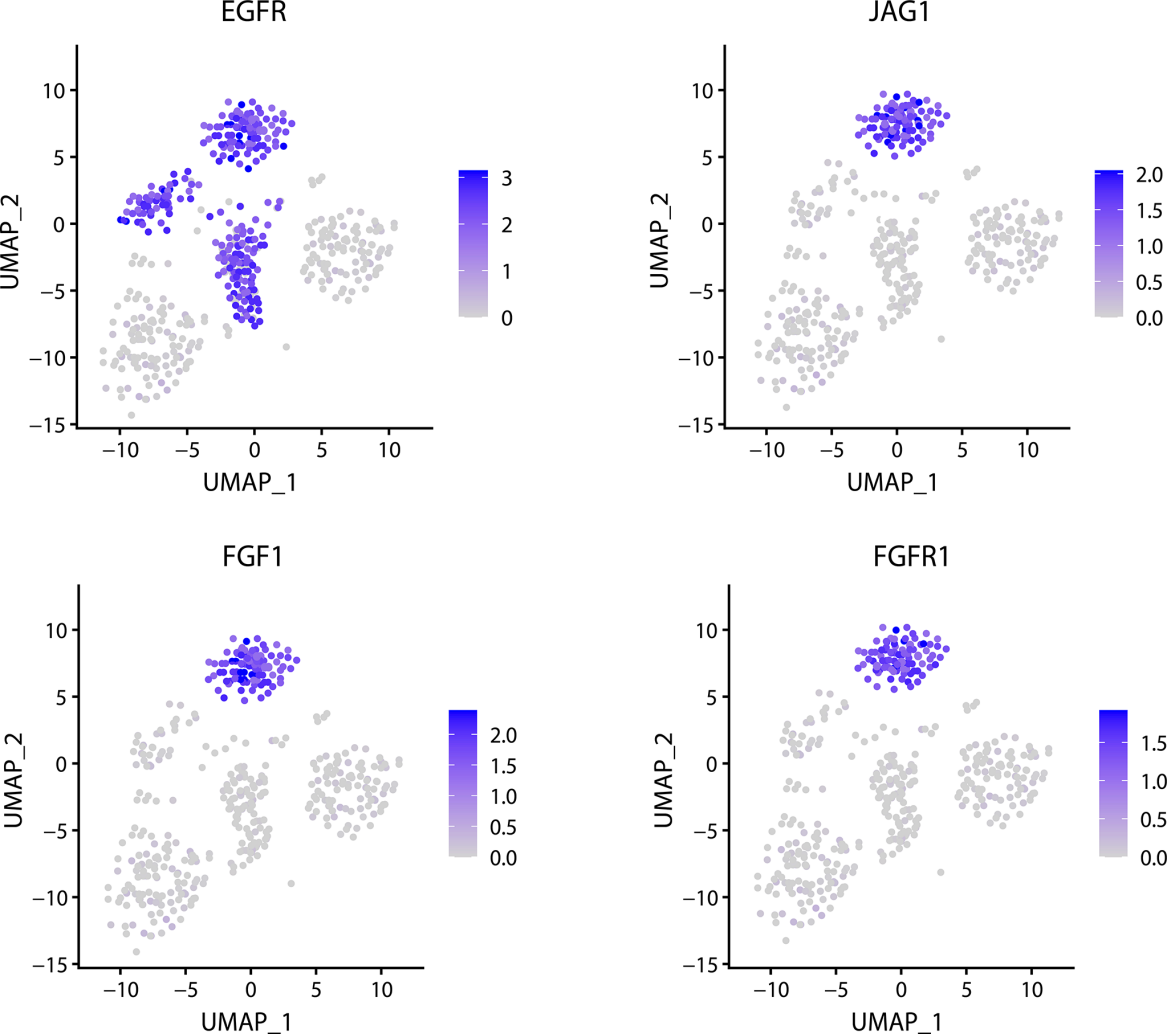
Related gene expression distribution in glioma data.

An important task of scRNA-seq analysis is to be able to identify the marker gene in the cluster and to determine whether the gene is a cell-specific maker gene. FEGFS can effectively identify the corresponding cell types from the glioma data and infer that EGFR is significantly expressed in the three tumors (**Figure 5**). Among them, the significant expression of EGFR is inversely correlated with the expression of PDGFRA in MGH30 cells. According to the experimental findings, the heterogeneous expression of RTKs and other signaling molecules across individual glioblastoma tumor cells may impair RTK signaling and the immunogenicity of targeted receptors.

## Discussion

In scRNA-seq data analysis, most of the existing methods use the whole single-cell gene expression matrix for analysis, without considering the influence of gene function from the perspective of molecular mechanism and ignoring certain biological significance.

In this study, we propose a novel scRNA-seq data analysis method based on gene function enrichment analysis to divide genes into different gene functional modules and to extract the characteristics of the cells from these functional feature matrices. As a data processing method, FEGFS considers the similarity between cells more fully, and it can improve the clustering accuracy. Our results suggest that gene function is indispensable for single-cell analysis like rare cell type inference and cell type identification.

It is of note that in the process of reducing the redundancy of the gene sets, we use two methods, namely, semantic similarity analysis and reduction of gene repetition between gene sets. We test the impact of these two methods on cell clustering. The results show that semantic similarity analysis does affect the performance of cell clustering, and although the effect of reduction of gene repetition is not obvious, it reduces the redundancy of gene sets and computational time complexity (**Table 2**) significantly. Especially in view of the increase in the size of the scRNA-seq dataset, a good data processing method with rapid operation speed is crucial.

**Table 2:** Comparison of running time.

Datasets	SC3	SSC	SIMLR	CORR	SinNLRR	Seurat	SSSC	FEGFS
***Biase***	49.38	2.58	1.51	2.72	4.19	4.00	6.72	2.10
***Goolam***	43.92	2.07	1.59	12.57	9.78	5.28	3.21	24.53
***Pollen***	48.78	3.08	4.35	59.24	18.41	4.18	1.51	23.76
***Patel***	57.92	2.13	5.77	52.27	15.51	3.90	2.23	5.40
***Klein***	608.09	99.00	452.00	3,180.00	2,976.60	37.88	147.00	989.55
***Zeisel***	615.77	76.00	362.00	7,740.00	6,381.90	127.89	144.00	1,710.15

Unit is in seconds.

However, FEGFS method still has a few limitations. Firstly, we need the gene ID in the scRNA-seq data to perform gene function enrichment analysis, but some scRNA-seq datasets do not provide gene ID, or the gene ID in the data cannot be matched, so these datasets cannot be considered, or the genes are deleted, which may result in the loss of some important information. Secondly, FEGFS is only combined with simple clustering method, which is not necessarily optimal. It is practicable to improve the clustering method after FEGFS analysis.

## Data Availability Statement

The original contributions presented in the study are included in the article/supplementary material. Further inquiries can be directed to the corresponding authors.

## Ethics Statement

Ethical review and approval was not required for the study on human participants in accordance with the local legislation and institutional requirements. The patients/participants provided their written informed consent to participate in this study.

## Author Contributions

JZ, JL, and JY conceived and proposed the method. CR and DR optimized the algorithm and designed the program. YL, DL, and LC analyzed the data. JZ and CR wrote the manuscript. All authors contributed to the article and approved the submitted version.

## Funding

This work is supported in part by the National Natural Science Foundation of China (Grant Nos. 61803065, 11971347), the Natural Science Foundation of Hunan province (No. 2018JJ2461), the Fundamental Research Funds for the Central Universities of China, and the project to introduce intelligence from oversea experts to the Changsha City (Grant No. 2089901).

## Conflict of Interest

JY and GT are currently employed by Genesis Beijing Co., Ltd.

The remaining authors declare that the research was conducted in the absence of any commercial or financial relationships that could be construed as a potential conflict of interest.

## Publisher’s Note

All claims expressed in this article are solely those of the authors and do not necessarily represent those of their affiliated organizations, or those of the publisher, the editors and the reviewers. Any product that may be evaluated in this article, or claim that may be made by its manufacturer, is not guaranteed or endorsed by the publisher.
